# Evaluation of primary health care nurses’ knowledge and neonatal screening performance for phenylketonuria in Alexandria

**DOI:** 10.1186/s12912-025-02719-4

**Published:** 2025-02-07

**Authors:** Esraa Mohammed Abd El-Samie Ismail, Wafaa Mohamed Elarousy, Shaymaa Saeed Mohamed Badawe, Rasha Mohamed Abohadida

**Affiliations:** 1 Faculty of Nursing, Alexandria University, Alexandria, Egypt; 2 Irbid National University, Irbid, Jordan; 3 Faculty of Nursing, King Salman International University, Sinai, Egypt

**Keywords:** Knowledge, Nurses, Performance, Phenylketonuria

## Abstract

**Introduction:**

Nurses play a crucial role in the primary prevention of phenylketonuria (PKU) within national screening programs for newborns. This is achieved through consult with the child’s provider promptly to arrange a PKU test, accurate collection of blood samples at maternal and child health centers, enable early detection of potential cases.

**Aim:**

This study aimed to assess the level of primary health care nurses’ knowledge and neonatal screening performance for phenylketonuria in Alexandria

**Methods:**

A descriptive research design was used. The study included all nurses (50 nurses) who are responsible for obtaining newborn screening test from 5 primary health care centers in Alexandria. Two tools were used to collect the necessary data: nurses’ knowledge regarding phenylketonuria disease assessment sheet and nurses’ performance about newborn screening for phenylketonuria observational checklist.

**Results:**

It was found that the mean age of participant was 42.94 ± 5.0 and 52% of them had completed their technical level of education and the means years of experience was 17.64 ± 4.84. The total mean score of nurses’ knowledge regarding phenylketonuria screening was 16.24 ± 1.99 with the mean percent score of 72.72 ± 8.27. It was also found the total score of nurses’ screening performance was 11.0 ± 0.97 with the mean percent score of 64.71 ± 5.70. A significant positive correlation was found between nurses’ knowledge and their screening performance with *P* = < 0.001.

**Conclusion:**

It was concluded that majority of participant nurses had correct knowledge in neonatal screening and many aspects of phenylketonuria disease. However, they still needed support in steps related to infection control.

**Supplementary Information:**

The online version contains supplementary material available at 10.1186/s12912-025-02719-4.

## Introduction

Phenylketonuria (PKU) is a rare inherited metabolic disorder in the phenylalanine (Phe) metabolism. It is caused by deficiency or absence of the phenylalanine hydroxylase enzyme. The normal function of phenylalanine hydroxylase is to catalyze the conversion of phenylalanine (Phe) to tyrosine (Tyr). This results in accumulation of abnormal amounts of Phe and reduction in Tyr in the blood [1]. The prevalence of PKU varies widely among ethnicities and geographic regions, affecting approximately 1 in 24,000 individuals worldwide. However, in the Middle East, some of the highest prevalence is in Egypt, Iran, and Jordan, where approximately 1:5,000 newborns are affected [2]. 

Phenylketonuria symptoms can manifest in varying degrees of severity. Infants diagnosed with PKU usually appear healthy at birth, with symptoms typically emerging around 6 months of age. If a child is untreated or inadequately treated, phenylalanine rises to toxic levels in the body especially in the brain causing infants to experience delays in motor skill development and exhibit lack of interest in their surroundings and subsequently significant neurodevelopmental sequelae including intellectual disability, mental retardation, seizures, learning disabilities, and emotional problems [3]. Phenylketonuria is diagnosed through performing serum Phe test between one and seven days after birth. Blood is obtained by pricking the heel of the newborn and analyzing it for phenylalanine concentration. Children with PKU need to keep Phe levels low. Therefore, keeping Phe levels between 120 and 360 μmol/L (2–6 mg/DL) for life is required [4]. 

Newborn screening plays a crucial role in identifying treatable disorders early, allowing for timely intervention and improved health outcomes. These screenings not only save lives but also enhance the quality of life for children and their families [5]. Worldwide, in most high-income countries, newborn screening generally exists as an efficient disease prevention system to reduce newborn morbidity and mortality. The Centers for Disease Control and Prevention (CDC) recognized newborn screening as one of the “Ten Great Public Health Achievements” during the first 10 years of the century. Furthermore, in 2015, the United Nations (UN) established 17 sustainable development goals. Newborn screening is one of the strategies to end preventable deaths of newborns and children under 5 years of age by 2030 in the third sustainable development goal [6]. In Egypt, 1996, the first newborn screening for metabolic disorders in Alexandria governorate took place through a study conducted by the Medical Research Institute where newborns were screened for three treatable inborn errors of metabolism which are phenylketonuria “PKU”, galactosemia and congenital hypothyroidism, aiming at early detection of these diseases and providing therapeutic regimens to guard against development of mental retardation. The results of this neonatal screening of 15,000 newborns showed a high frequency of Phenylketonuria (1:7000), and of hypothyroidism (1:3000), which convinced the health authorities in Egypt to start a mass neonatal screening program for PKU and hypothyroidism in all Egyptian governorates in 2003 [7, 8]. 

In 2023, an infographic showcasing the accomplishments of the President of the Republic’s program for early detection of genetic diseases in newborns was released by The Ministry of Health and Population on its official Facebook page. The screening is carried out by blood sample analysis in cooperation with the Egyptian Centre for Diseases and Control (ECDC), followed by a second test to confirm the diagnosis if the first comes back positive [9]. 

Dietary management and pharmacologic treatment are essential for infants with PKU. Pharmacological treatments such as enzyme replacement therapy through viral delivery of protein fusions targeting the liver as well as enzyme substitution therapy [10]. Dietary restriction of phenylalanine remains to be the mainstay of treatment for PKU. It should be instituted as soon as the diagnosis is confirmed and must be commenced from the neonatal period and adhered to for life [11]. The cornerstone for PKU dietary regimen is typically low in high protein [12, 13]. It also may include specially formulated medical foods and protein substitutes that are phenylalanine-free or low in phenylalanine [14, 15]. It is recommended that the child’s diet is well-planned to include a variety of fruits and vegetables to prevent micronutrient deficiencies commonly associated with restrictive diets [16]. Infants with PKU need a special formula that contain high protein and low phenylalanine. It can be used throughout life as a protein source that is extremely low in phenylalanine and balanced for the remaining essential amino acids [17]. 

Primary health care nurses play a crucial role in the primary prevention of PKU within national screening programs for newborns. This is achieved through consult with the child’s provider promptly to arrange a PKU test. Accurate collection of blood samples at maternal and child health centers enables early detection of potential cases. It is crucial for the nurse to be well-versed in obtaining accurate test results, ensuring that the baby has consumed protein from breast milk or formula before the blood sample is drawn [17]. In addition, nurses must adhere to neonatal screening procedure as improper heel-stick technique can damage the structures of the foot, including the calcaneus bone and soft tissues. In fact, some reports have documented difficulties walking later in life. It is safe to perform a heel stick if the puncture site is limited to the medial and lateral planter aspects of heel pad [18]. 

Nurses are at the forefront of identifying cases and providing essential health education on the disease and dietary requirements, as well as ensuring proper specimen recording and analysis. It is imperative for PHC nurses to have a comprehensive understanding of PKU disease, screening procedures, sampling techniques, and overall managing responsibilities within their respective centers and units.

## Significance of the study and research gap

Part of Egypt’s vision 2030 focuses on the rights of all Egyptians for healthy life with healthcare system capable of improving health conditions through early intervention, and preventive coverage. Nurses have many important roles in primary healthcare centers such as giving vaccination, assessing growth measurements, performing newborn screening test, and instructing parents about the benefit of early diagnoses in preventing complications of PKU. They play an important role in early detection through being competent in adherence to neonatal screening procedure. Early detection is the key to manage phenylketonuria as the infants appear healthy at birth, and if untreated or inadequately treated, infants may experience developmental delays and intellectual disabilities that can affect the children’s entire life, children’s families, and the community. Overall, research in this area plays a crucial role in ensuring that primary health care nurses are equipped with the necessary knowledge and skills to effectively contribute to neonatal screening programs, thereby improving outcomes for newborns and their families. So, the aim of this study is to evaluate the primary health care nurses’ knowledge and neonatal screening performance for phenylketonuria in Alexandria.

## Aim

The aim of this study is to evaluate the primary health care nurses’ knowledge and neonatal screening performance for phenylketonuria.

## Objectives

1. Assess primary health care nurses’ knowledge regarding phenylketonuria (PKU) and newborn screening test.
2. Evaluate primary health care nurses’ performance for phenylketonuria neonatal screening.

### Research question

What is the primary health care nurses’ knowledge and neonatal screening performance for phenylketonuria?

## Design

A descriptive research design was used to accomplish this study.

### Settings

The study was conducted at 5 primary healthcare centers in Alexandria. Dana, El-seyouf, Smouha, El -Hadrah Qebli, and El -Agamy family health centers.

### Subjects

**Fig. 1:**
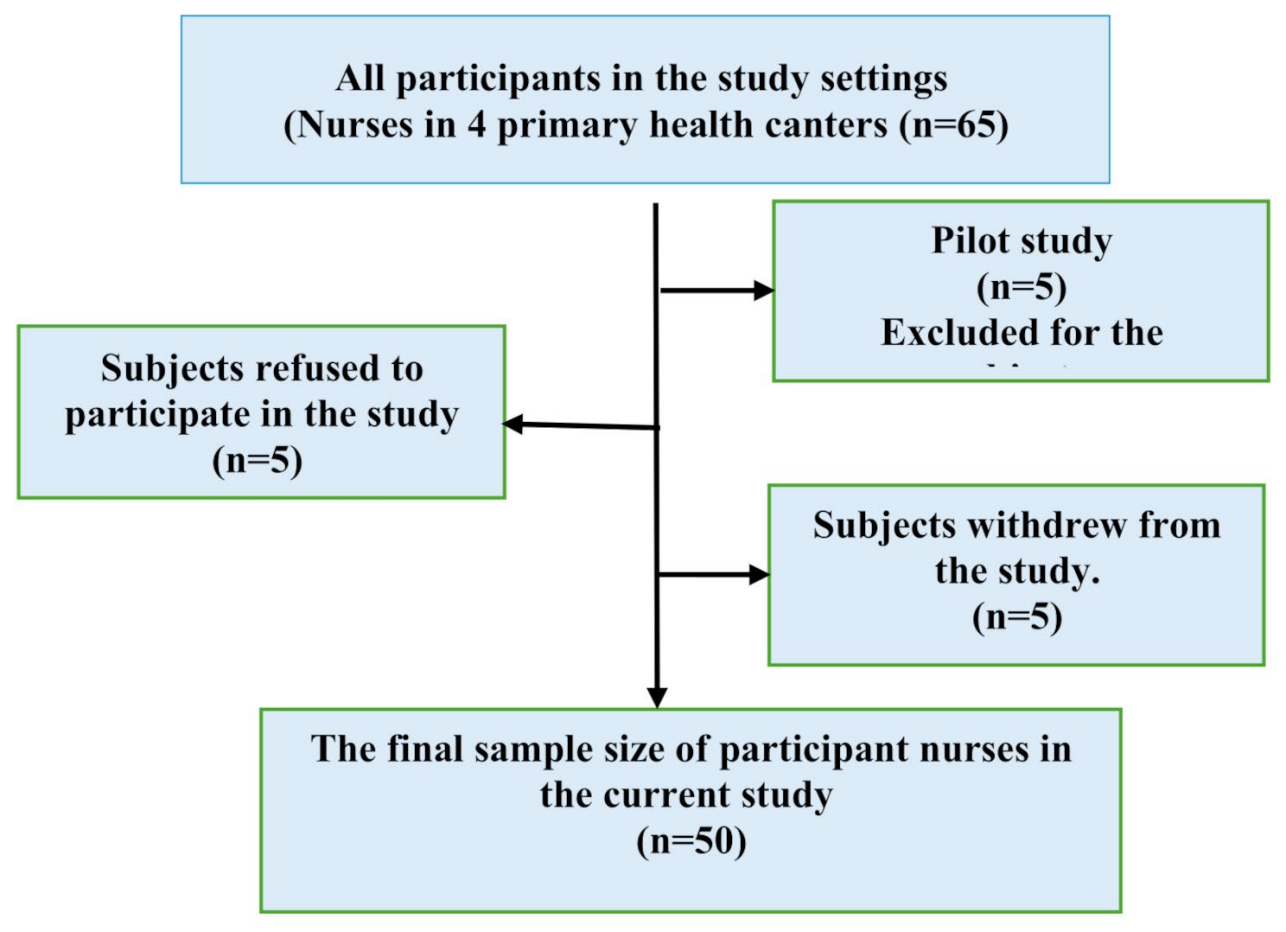
Sampling flow chart

All nurses (65 nurses) who are responsible for obtaining newborn screening test for phenylketonuria in the primary health care centers mentioned above were invited to participate in the study, 5 of them refused to participate and another 5 withdrew from the study. Fifty nurses participated in the study as follow: 8 nurses from Dana, 8 nurses from El-seyouf, 9 nurses from Smouha, 11 nurses from El-Hadrah Qebli and 14 from El-Agamy family health centers (Fig. 1).

### Study tools

Two tools were used to collect the necessary data. **The tools were developed by the researchers** after reviewing of recent and relevant literatures [19, 20]. 

#### Tool I: nurses’ knowledge regarding phenylketonuria disease assessment sheet

It is divided into 3 parts. Part 1 included nurses’ characteristics such as age, educational level, years of experiences and attendance of training courses/lectures or discussion regarding newborn screening program for phenylketonuria. Part 2 included nurses’ knowledge about phenylketonuria disease such as definition, causes, symptoms and signs, diagnosis, prevention of potential complications, and different methods of treatment (dietary restriction, special medical formula and medical treatment in addition to available community services). Part 3 included nurses’ knowledge about phenylketonuria screening test regarding timing, benefits and follow up of tests. Nurses’ knowledge in part 2 and 3 was scored as the follows: 1 for correct and complete answer, and zero for unknown or wrong answer.

#### Tool II: nurses’ performance about newborn screening for phenylketonuria observational checklist

This tool was used to assess nurses’ performance before, during, and after newborn screening for phenylketonuria. Performance was scored as 1 for correct steps and zero for incorrect or not done steps.

### Method

1. Approval from the Research Ethics Committee of the Faculty of Nursing at Alexandria University was obtained.
2. Official letter was directed to the responsible authority of selected family health centers to facilitate research implementation after explaining the aim of the study.
3. Tool I, II were developed by the researchers after thorough reviewing of recent and relevant literatures.
4. Tools were tested for its content validity by five experts in the field of pediatric and community health nursing.
5. Reliability of the tools was done using Cronbach Coefficient Alpha test where *r* = 0.866 for tool I and 0.848 for tool II.
6. A pilot study was carried out on 10% nurses (5 nurses) to test the clarity and feasibility of the tools. Those nurses were excluded from the study subjects.
7. Nurse was observed individually by the researcher once during newborn screening using tool II.
8. Then, nurses were interviewed individually to assess their knowledge using tool I.
9. Data were collected over a period of from September 2022 to July 2023.
10. After completion of data collection, the necessary statistical analysis was used.

### Ethical considerations

Witness informed consent was obtained from the head nurses of all selected primary health care centers after explaining the aim of the study for tool II. A written informed consent was obtained from all participant nurses after explanation of the aim of the study and assurance that the collected data is used only for the study purposes. Study subjects’ anonymity is maintained as well as confidentiality of the collected data. Subjects’ voluntary participation and their right to withdraw from the study at any time without penalty were assured. Dealing with the study subjects was based on mutual respect with no discrimination.

### Statistical analysis

Data were fed to the computer and analyzed using IBM SPSS software package version 23.0. One way ANOVA test was used to compare between more than two categories. Student t-test was used to compare two categories for normally distributed quantitative variables. Pearson coefficient was used to correlate between normally distributed quantitative variables. Significance of the obtained results was judged at the 5% level.

## Results

Table 1 displays the distribution of the participant nurses according to their demographic characteristics. Concerning the number of nurses in family health centers; it was found that more than one quarter (28%) of participant nurses were from the El Agamy family health center followed by more than one fifth (22%) of nurses in El Hadra Qebli. Slightly less than one fifth (18%) of participant nurses were from Smouha and an equal percent of nurses (16%) were from Danna and Seyouf family health care centers.

Additionally, it was found that nearly half of participant nurses’ (48%) aged from 41 to 50 years old, with a mean age of 42.94 ± 5.0. Considering educational level, it was observed that 52% of participant nurses had completed their technical level of education and slightly more than two thirds of them (68.0%) had more than 15 years of experience with means experience years of 17.64 ± 4.84.

Regarding attending training courses about newborn screening program for Phenylketonuria. It was found that less than two-thirds (62%) of participant nurses attended training courses, and more than half of them obtained two training courses. Moreover, it was noticed that two days training course was reported by 51.6% of them.

Table 2 highlights the nurses’ knowledge regarding Phenylketonuria. It was revealed that, all participant nurses had correct knowledge about the definition, diagnosis, age of starting treatment, types of allowed foods, and time and Benefits of neonatal screening test. In addition, the majority had correct knowledge about degree of PKU severity, how long should the diet be used, types of allowed formula, giving breast milk, and available community services that support PKU children ( 82%, 84%, 84%, 94% & 94% respectively). On the other hands, the participant nurses had incorrect knowledge about blood level of PKU to be achieved during the treatment (88%), name of missing enzyme (74%) and management of PKU (62%). Moreover, it was clear from the table that the total mean score of nurses’ knowledge regarding Phenylketonuria screening was 16.24 ± 1.99 and the mean percent score was 72.72 ± 8.27.

Table 3 portrays the nurses’ screening performance for Phenylketonuria. It was revealed that, all participant nurses correctly performed the following steps: selection of puncture area, surface area to puncture, puncture heel at a 90-degree angle, seal blood containers, apply direct pressure on puncture site, label collection tubes and dispose of puncture device in sharps container. While the majority of them performed the following steps incorrectly: perform hand hygiene, administrate 25% sucrose’s, and remove gloves (90, 80, 84% respectively). In addition, all the participant nurses did not perform hand hygiene after screening test. It was clear from the table that the total score of nurses’ practice regarding Phenylketonuria was 11.0 ± 0.97 and the mean percent score of practice was 64.71 ± 5.70.

Table 4 illustrates the correlation between nurses’ knowledge and screening performance for Phenylketonuria. A significant positive correlation was found between nurses’ knowledge and practice (*P* < 0.001).

Table 5 presents the relationship between nurses’ knowledge and screening performance for phenylketonuria with their characteristics. The result revealed that there were highly statistically significant relationships between the total mean score of the nurses’ knowledge with their level of education, years of experiences and attending training courses with *P* value of < 0.001. Moreover, a highly statistically significant differences were found between mean score of nurses’ screening performance and their age, level of education, years of experiences and attending training courses with *P* value of < 0.001, 0.003, < 0.001 and < 0.001 respectively.

Table 6 displays the effect of nurses’ characteristics on knowledge and practice through multiple linear regression analysis. It was found that significant relationships were found between the units, level of education, attendance training courses, and their nurse’s knowledge (*P* = 0.044, 0.032, < 0.001, and 0.003, respectively). On the other hand, the same table found significant relationships between the attendance training courses and nurses’ practice (*P* = 0.045). 

**Fig. 2:**
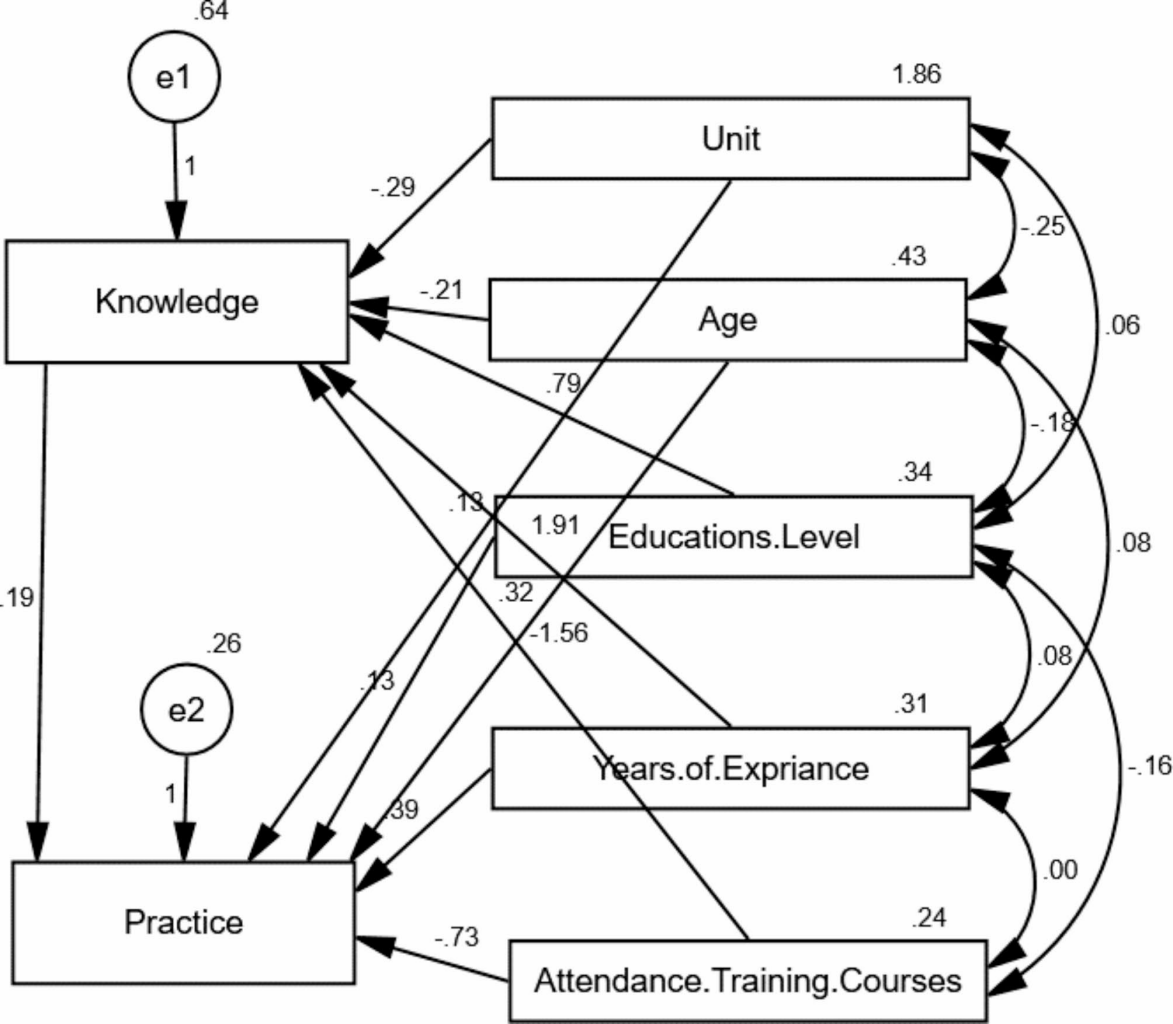
Structure Equation Modeling (*n* = 50) Model fit parameters CFI; IFI; RMSEA (1.000; 1.000; 0.104) CFI = Comparative fit index; IFI = incremental fit index; and RMSEA = Root Mean Square Error of Approximation Model χ^2^; significance 12.042* (< 0.001*)

Figure 2 shows that demographic characteristics have a direct significant effect on nurses’ knowledge and practice, and indirect significant effect on practice mediated by knowledge. Tables 7 and 8 show the direct and indirect effects of study variables on nurses’ knowledge and practices. It was found that there were direct and significant relationships between nurses’ knowledge and the unit, level of education, years of experience and attendance training courses (*P* = 0.029, 0.019, < 0.001, and < 0.001, respectively). Moreover, it was revealed that there were direct and significant relationships between nurses’ practice and their knowledge, and their attendance training courses (*P* = 0.033, and 0.027, respectively).

**Table 1:** Distribution of participant nurses according to their demographic characteristics (*n* = 50)

Nurses’ characteristics	No.	%
**Family Health Centers**		
Danna	8	16.0
El Agamy	14	28.0
El Seyouf	8	16.0
El Hadara Qebli	11	22.0
Smouha	9	18.0
**Age (years)**		
30–40	20	40.0
41–50	24	48.0
> 50	6	12.0
**Mean ± SD**	**42.94 ± 5.0**	
**Level of education**		
Diploma	24	48.0
Technical	26	52.0
**Years of experience**		
< 10	2	4.0
10 - <15	14	28.0
≥ 15	34	68.0
Mean ± SD	17.64 ± 4.84	
**Attendance training courses about newborn screening program for Phenylketonuria**		
Yes	31	62.0
No	19	38.0
**Number of training courses (n = 31)**		
Once	13	41.9
Two	18	58.1
**Duration of each course (n = 31)**		
One day	15	48.4
Two days	16	51.6

**Table 2:** The nurses’ knowledge levels regarding Phenylketonuria screening and disease (*n* = 50)

Nurses knowledge regarding phenylketonuria	Incorrect answer		Correct answer	
	No.	%	No.	%
- Definition of Phenylketonuria (PKU)	0	0.0	50	100.0
- Causes of PKU	11	22.0	39	78.0
- Clinical manifestation of PKU	14	28.0	36	72.0
- Name of missing enzyme is in PKU	37	74.0	13	26.0
- Early clinical manifestation that appear in neonates PKU	16	32.0	34	68.0
- Management of PKU	31	62.0	19	38.0
- Potential complications of PKU	29	58.0	21	42.0
- Age of starting treatment of PKU	0	0.0	50	100.0
- Age of start PKU symptoms	14	28.0	36	72.0
- Diets of PKU	27	54.0	23	46.0
- Degree of severity of PKU	9	18.0	41	82.0
- Types of restricted foods	13	26.0	37	74.0
- How long should the diet be used in PKU	8	16.0	42	84.0
- Types of allowed foods	0	0.0	50	100.0
- Types of allowed formula	8	16.0	42	84.0
- Diagnosis of PKU	0	0.0	50	100.0
- Give breast milk to a PKU infant	3	6.0	47	94.0
- Normal level of Phenylketonuria in the blood	21	42.0	29	58.0
- Level of Phenylketonuria in the blood to be achieved during the treatment	44	88.0	6	12.0
- Time of neonatal screening test	0	0.0	50	100.0
- Benefits of neonatal screening test	0	0.0	50	100.0
- Available community services that support PKU children	3	6.0	47	94.0
**Total mean score of knowledge**	**16.24 ± 1.99**			
**Mean percent score of knowledge**	**72.72 ± 8.27**			

**Table 3:** The nurses’ screening performance for phenylketonuria (*n* = 50)

Nurses practice regarding Phenylketonuria	Not done		Done	
	No.	%	No.	%
- Preform hand hygiene and clean blue tray	45	90.0	5	10.0
- Collect equipment. Select the appropriate puncture device	5	10.0	45	90.0
- Administrate 25% sucrose’s and provide comfort measure	40	80.0	10	20.0
- Preform hand hygiene apply gloves and prepare equipment	29	58.0	21	42.0
- Nominate an area for puncture on the foot on medial lateral plantar surface	0	0.0	50	100.0
- Select the surface area to puncture. Continue in a stepping ladder pattern from the first puncture for subsequent blood sampling	0	0.0	50	100.0
- Clean foot with alcohol and allow 30 s to dry completely	36	72.0	14	28.0
- Puncture heel holding the puncture device at a 90-degree angle	0	0.0	50	100.0
- Wipe away fist drop of blood with gauze	21	42.0	29	58.0
- Collect blood in correct order of draw, gently agitating tubes between each drop while avoiding scraping and scooping	29	58.0	21	42.0
- Seal blood containers	0	0.0	50	100.0
- Apply direct pressure on puncture site	0	0.0	50	100.0
- Label collection tubes with correct	0	0.0	50	100.0
- Dispose of puncture device in sharps container	0	0.0	50	100.0
- Collect and dispose of remaining equipment	3	6.0	47	94.0
- Clean blue tray and remove gloves	42	84.0	8	16.0
- Preform hand hygiene after test	50	100.0	0	0.0
**Total mean score of screening performance**	**11.0 ± 0.97**			
**Mean percent score of screening performance**	**64.71 ± 5.70**			

**Table 4:** Correlation between nurses’ knowledge and screening performance for phenylketonuria (*n* = 50)

	*R*	*p*
Nurses’ knowledge and practice	0.764*	< 0.001*

r: Pearson coefficient

*: Statistically significant at *p* ≤ 0.05

**Table 5:** Relationship between Nurses knowledge and Screening Performance for Phenylketonuria with their characteristics (*n* = 50)

Nurses’ characteristics	Knowledge	Practice
**Age (years)**		
30–40	15.50 ± 1.54	10.40 ± 0.68
41–50	16.63 ± 2.16	11.29 ± 1.0
> 50	17.17 ± 2.04	11.83 ± 0.41
**F(p)**	**2.664 (0.080)**	9.668* (< 0.001*)
**Level of education**		
Diploma	14.96 ± 2.10	10.58 ± 1.06
Technical	17.42 ± 0.81	11.38 ± 0.70
**t(p)**	5.404* (< 0.001*)	3.131* (0.003*)
**Years of experience**		
< 10	13.0 ± 0.0	10.0 ± 1.41
10 - <15	14.57 ± 1.60	10.21 ± 0.89
≥ 15	17.12 ± 1.49	11.38 ± 0.74
**F(p)**	18.931* (< 0.001*)	12.075* (< 0.001*)
**Attendance training courses about newborn screening program for Phenylketonuria**		
Yea	17.19 ± 1.22	11.48 ± 0.68
No	14.68 ± 2.03	10.21 ± 0.85
**t(p)**	4.876* (< 0.001*)	5.838* (< 0.001*)

F: One way ANOVA test t: Student t-test *: Statistically significant at *p* ≤ 0.05.

**Table 6:** Multiple Linear Regression Analysis Showing the Effect of Nurses characteristics on knowledge and practice (*n* = 50)

Nurses’ characteristics	Knowledge						Practice					
	B	Beta	t	**p**	95% CI		B	Beta	t	**p**	95% CI	
					**LL**	**UL**					**LL**	**UL**
Age (years)	-0.208	-0.070	-0.721	0.475	-0.787	0.372	0.318	0.221	1.714	0.094	-0.056	0.693
Level of education	0.787	0.200	2.215*	0.032*	0.071	1.504	0.134	0.070	0.556	0.581	-0.351	0.619
Years of experience	1.915	0.543	7.117*	< 0.001*	1.373	2.457	0.390	0.227	1.541	0.131	-0.120	0.901
Attendance training courses (No)	-1.555	-0.384	-3.150*	0.003*	-2.550	-0.560	-0.725	-0.367	-2.068*	0.045*	-1.432	-0.018
Knowledge	-	-	-	-	-	-	0.193	0.395	1.994	0.052	-0.002	0.388
R^2^ = 0.835, Adjusted R^2^ = 0.816, F = 44.450^*^,*p* < 0.001^*^							R^2^ = 0.721, Adjusted R^2^ = 0.682, F = 18.502^*^,*p* < 0.001^*^					

F, p: f and *p* values for the model

R^2^: Coefficient of determination

B: Unstandardized Coefficients

Beta: Standardized Coefficients

t: t-test of significance

LL: Lower limit UL: Upper Limit

*: Statistically significant at *p* ≤ 0.05

**Table 7:** Direct and indirect effect of the study variables on nurses’ knowledge and their practices

Variable 1		Variable 2	Standardized regression weights	S.E	C.*R*	*p*-value
Knowledge	←	Unit	-0.215	0.131	-2.188*	0.029*
Knowledge	←	Age (years)	-0.075	0.273	-0.761	0.447
Knowledge	←	Level of education	0.251	0.337	2.338*	0.019*
Knowledge	←	Years of experience	0.586	0.255	7.511*	< 0.001*
Knowledge	←	Attendance training courses	-0.415	0.468	-3.324*	< 0.001*
Practice	←	Knowledge	0.369	0.091	2.129*	0.033*
Practice	←	Unit	0.183	0.087	1.469	0.142
Practice	←	Level of education	0.081	0.225	0.593	0.553
Practice	←	Years of experience	0.228	0.237	1.645	0.100
Practice	←	Attendance training courses	-0.370	0.329	-2.207*	0.027*
Practice	←	Age (years)	0.220	0.174	1.830	0.067

**Table 8:** Direct and indirect effect of study variables on nurses’ knowledge and their practices

Variables		Direct effect	Indirect effect	CI			*p*-value
Knowledge	Unit	-0.286	0.0	-0.322	-	-0.250	0.029*
Knowledge	Age (years)	-0.208	0.0	-0.268	-	0.148	0.447
Knowledge	Level of education	0.787	0.0	0.732	-	0.842	0.019*
Knowledge	Years of experience	1.915	0.0	1.873	-	1.957	< 0.001*
Knowledge	Attendance training courses	-1.555	0.0	-1.682	-	-1.428	< 0.001*
Practice	Knowledge	0.193	0.0	0.136	-	0.250	0.033*
Practice	Unit	0.128	-0.055	-0.074	-	0.182	0.142
Practice	Level of education	0.134	0.152	-0.082	-	0.186	0.553
Practice	Years of experience	0.390	0.369	-0.048	-	0.438	0.100
Practice	Attendance training courses	-0.725	-0.300	-0.788	-	-0.662	0.027*
Practice	Age (years)	0.318	-0.040	-0.090	-	0.447	0.067

## Discussion

Phenylketonuria is a genetic condition caused by a defect in gene that helps produce the phenylalanine hydroxylase enzyme required for the breakdown of phenylalanine that results in rising levels of phenylalanine in blood and tissues. Elevating phenylalanine to dangerous levels in the body can lead to serious complications in children, especially mental retardation. Newborn screening identifies these children to start proper treatment and dietary management early which improves their quality of life [21]. 

The current study found that more than two-thirds of nurses attended training courses, and more than half of them obtained at least two training courses for two days of each course. This result could be related to the fact that continuous education and training programs established by the Ministry of Health and Population enhance nurses’ knowledge and practices. It is also reflected in the attention of decision-makers to train the nurses about the national neonatal screening program for their crucial role in obtaining the screening test and to counsel and educate the families about the importance of the screening and the targeted diseases.

The finding of the present study revealed that the nurses’ total mean score of knowledge regarding phenylketonuria was 16.24 ± 1.99 and the mean percent score of knowledge was 67.67 ± 8.27. These results might be related to the availability of educational training for nurses which updated knowledge regarding phenylketonuria screening. This result is congruent with the study findings of **Jose & Manalo **,**2023** [22] who found that providing pediatric residents with an educational module resulted in significantly improved knowledge of neonatal screening. The study also highlighted the necessity to continuously educate healthcare workers on all aspects of neonatal screening so that they can in turn educate parents. On the other side, the finding of **Joseph**,** 2017** [23] who studied the newborn screening challenges for NICU nurses, reported that NICU nurses face several challenges with the expansion of newborn screening programs and gain knowledge to answer questions posed by empowered parents and educate them appropriately.

Regarding nurses’ practice toward the phenylketonuria screening test in this study, the nurses’ total score of practice regarding phenylketonuria was 11.0 ± 0.97 and the mean percent score of practice was 64.71 ± 5.70. The results revealed that all nurses correctly performed all participant nurses correctly performed a selection of puncture surface area, and heel at right angle, seal blood containers, apply direct pressure on puncture site, label collection tubes and dispose of puncture device correctly. These findings might be related to continue supervision and training courses for participant nurses that improved their performance in the steps of selection of a suitable site and withdrawal of a sample. Surprisingly, the majority of nurses did not perform the steps related to infection control correctly, such as performing hand hygiene before and after the screening test, cleaning the blue tray, and removing gloves, even though the hand hygiene facilities are available to all of them in all family health centers. This may be attributed to the overload of nurses with many neonates each day of screening, lack of knowledge about the importance of hand hygiene and limited doing it to the beginning and end of the day.

Moreover, the findings of the current study showed that most nurses had not performed correctly regarding administering 25% sucrose and providing comfort measures for pain management to the neonates. This may be attributed to the fact that nurses do not realize that screening procedures are painful procedures, and the neonates feel pain from them. The study finding of **Khaton**,** 2016** [24] supports the current study and cites that only 16% of participant nurses know 9–13 steps of the PKU screening procedure correctly. On the other side, the study finding of **Venegas et al.**,** 2019** [25], who studied the barriers and facilitators to using pain treatment during newborn screening blood tests at a mother-baby unit, contradicts this study and cites that 53% of nurses used sucrose and 44% of them reported breastfeeding was supported in pain management.

The findings of the current study revealed that a significant positive correlation was detected between nurses’ knowledge and practice. This finding could be related to the fact that knowledge is needed to support the practice in addition to continuous training courses for participant nurses to improve nurses’ knowledge and practices. The study findings of **Said** & **Draz**, **2019** [26] were congruent with the present study and cited that there was a statistically significant positive correlation between total knowledge of the participant nurses and total practice towards the management of children with phenylketonuria.

In addition, the findings of the present study revealed that there were direct and significant relationships between nurses’ practice and their knowledge with their attendance training courses. This finding related to the fact that continuous education updated their nurses’ knowledge and improved their skills regarding neonatal screening performance for phenylketonuria. The study finding of **Said** & **Draz**, **2019** [26] was in the same line with the present study and cited that there is a positive statistical relationship between nurses’ knowledge and practice post-program implementation.

Furthermore, the results of this study found that the age of the participating nurses affected positively their knowledge and was statistically significant with their practice (*P* = 0.080 and < 0.001, respectively). Moreover, the results of the current study revealed that there were significant relationships between nurses’ knowledge and practices with their level of education (*P* = < 0.001, 0.003, respectively) and years of experience. These findings might be related to the fact that nurses’ age, their educational level, and years of experience were considered as factors affecting their knowledge and practice level during neonatal screening performance for phenylketonuria. The study findings of **Khedr et al.**,** 2022** [27] were consistent with the findings of the current study and mentioned that there was a statistically significant difference between the participant nurses’ total level of practice and their age, educational level, and years of experience.

### Study limitation

Although each study nurse was secured that all gathered data is used for research purposes only. Five nurses refused to participate, and 5 others withdrew during the study thinking that it would negatively impact their image. In addition, the study was conducted at only five primary health care centers with a limited sample size of 50 nurses which might limit the generalization of the results.

### Conclusion and recommendations

It was concluded from this current study that majority of participant nurses had correct knowledge in neonatal screening and many aspects of phenylketonuria disease. However, they still needed support in steps related to infection control. A periodical educational and e-learning programs for nurses about the neonatal screening and infection control are recommended.

### Practice implications

The identification of gaps either in knowledge or screening performance and highlighting areas where further training is needed. This can be achieved by continuous training for primary health care nurses to enhance the overall accuracy and efficiency of neonatal screening programs for PKU.

## Electronic supplementary material

Below is the link to the electronic supplementary material.

Supplementary Material 1

## Data Availability

The datasets used or analyzed in this study are available from the corresponding author upon request.

## References

[1] Casey L. Caring for children with phenylketonuria. Can Family Physician Medecin De Famille Canadien. 2013;59(8):837–40. 23946023 PMC3743692

[2] Elhawary A, AlJahdali A, Abumansour S, Elhawary N, GaboonN, Dandini M, Madkhali A, Alosaimi W, Alzahrani A, AljohaniF, Melibary M, Kensara A. Genetic etiology and clinical challenges of phenylketonuria. Hum Genomics. 2022;16(1):22. 10.1186/s40246-022-00398-9 35854334 10.1186/s40246-022-00398-9 PMC9295449

[3] Ashe K, Kelso W, Farrand S, Panetta J, Fazio T, De Jong G, Walterfang M. Psychiatric and cognitive aspects of phenylketonuria: the limitations of diet and promise of new treatments. Front Psychiatry. 2019;10:561. 10.3389/fpsyt.2019.0056 31551819 10.3389/fpsyt.2019.00561 PMC6748028

[4] Van Spronsen J, Blau N, Harding C, Burlina A, Longo N, Bosch M. Phenylketonuria. Nat Reviews Disease Primers. 2021;7(1):36. 10.1038/s41572-021-00267-0 34017006 10.1038/s41572-021-00267-0 PMC8591558

[5] Ding S, Han L. Newborn screening for genetic disorders: current status and prospects for the future. Pediatr Invest. 2022;6(4):291–8. 10.1002/ped4.12343 10.1002/ped4.12343 PMC9789938 36582269

[6] Therrell BL, Padilla CD, Borrajo GJC, Khneisser I, Schielen PCJI, Knight-Madden J, Malherbe HL, Kase M. Current status of newborn bloodspot screening worldwide 2024: a comprehensive review of recent activities (2020–2023). Int J Neonatal Screen. 2024;10(2):38. 10.3390/ijns10020038 38920845 10.3390/ijns10020038 PMC11203842

[7] El-Attar A, Elkaffas M, Aglan A, Naga S, Nabil A, Abdallah Y. Genomics in Egypt: current status and future aspects. Front Genet. 2022;13:797465. 10.3389/fgene.2022.797465 35664315 10.3389/fgene.2022.797465 PMC9157251

[8] Temtamy A. The development of human genetics at the National Research Centre, Cairo, Egypt: a story of 50 years. Annu Rev Genom Hum Genet. 2019;20(1):1–19. 10.1146/annurev-genom-083118-015201 10.1146/annurev-genom-083118-015201 30848958

[9] State Information Services (SIS). Newborns examined as part of Egypt’s initiative for early detection of genetic diseases; Egypt Today: 2023. Available at: 267K newborns examined as part of Egypt’s initiative for early detection of genetic diseases - EgyptToday.

[10] Viecelli M, Harbottle P, Wong P, et al. Treatment of phenylketonuria using minicircle-based naked-DNA gene transfer to murine liver. Hepatology. 2014;60(1):1035–43. 24585515 10.1002/hep.27104 PMC4449723

[11] Al Hafid N, Christodoulou J, Phenylketonuria. A review of current and future treatments. Translational Pediatr. 2015;4(4):304–17. 10.3978/j.issn.2224-4336.2015.10.07 10.3978/j.issn.2224-4336.2015.10.07 PMC4728993 26835392

[12] MacDonald A, van Wegberg J, Ahring K, Beblo S, Bélanger-Quintana A, Burlina A, Campistol J, Coşkun T, Feillet F, Giżewska M, Huijbregts C, Leuzzi V, Maillot F, Muntau C, Rocha C, Romani C, Trefz F, van Spronsen J. PKU dietary handbook to accompany PKU guidelines. Orphanet J Rare Dis. 2020;15(1):171. 10.1186/s13023-020-01391-y 32605583 10.1186/s13023-020-01391-y PMC7329487

[13] Russo L, Puleo S, Cavella S, Scala I, Fidaleo M, Di Monaco R. Advancements in food science for phenylketonuria (PKU) management: a comprehensive review. Crit Rev Food Sci Nutr. 2024:1–15. 10.1080/10408398.2024.2360075 10.1080/10408398.2024.2360075 38818634

[14] Newby C. Introducing a granule-based protein substitute to the diet of a child with phenylketonuria to address reluctance to ingest phenylalanine-free protein substitute: a case report. Nutr Health. 2024;30(1):35–8. 10.1177/02601060231184934 37365866 10.1177/02601060231184934 PMC10924697

[15] Maler V, Goetz A, El Khalil P, Meunier F, Maillot F. Aspartame and phenylketonuria: an analysis of the daily phenylalanine intake of aspartame-containing drugs marketed in France. Orphanet J Rare Dis. 2023;18(1):142. 10.1186/s13023-023-02770-x 37291632 10.1186/s13023-023-02770-x PMC10249154

[16] Van Wegberg J, MacDonald K, Ahring A, Bélanger-Quintana N, Blau M, Bosch A, Burlina J, Campistol F, Feillet M, Giżewska, et al. The complete European guidelines on phenylketonuria: diagnosis and treatment. Orphanet J Rare Dis. 2017;12(1):162. 10.1186/s13023-017-0685-2 29025426 10.1186/s13023-017-0685-2 PMC5639803

[17] Singh H, Rohr F, Frazier D, Cunningham A, Mofidi S, Ogata B, Splett L, Moseley K, Huntington K, Acosta B, Vockley J, Van Calcar C. Recommendations for the nutrition management of phenylalanine hydroxylase deficiency. Genet Med. 2014;16(2):p121–31. 10.1038/gim.2013.179 PMC3918542 24385075

[18] Ibrahem Z, Ouda W, Bayoumi O. Assessment of nurses’ performance for pain management of neonates undergoing heel stick puncture. Egypt J Health care. 2024;15(1).

[19] Borrajo G. Newborn screening for phenylketonuria: Latin American consensus guidelines. J Inborn Errors Metabolism& Screen. 2016;4:1–5. 10.1177/2326409816682764

[20] Öztürk F, et al. Assessment of parents´ knowledge regarding phenylketonuria and its affecting factors: a cross-sectional study. Pan Afr Med J. 2022;41(308). 10.11604/pamj.2022.41.308.25936 10.11604/pamj.2022.41.308.25936 PMC9250662 35855041

[21] Baraka M, Gouda S. Upgrading mothers’ knowledge and practice regarding the care of their children suffering from phenylketonuria. Egypt J Health Care. 2022;13(1).

[22] Jose P, Manalo E. Newborn screening knowledge, attitudes and practices among obstetrics-gynecology residents, pediatric residents, and newborn screening nurses in a Tertiary Government Hospital in the Philippines during the COVID-19 pandemic. J Neonatal Screen. 2023;9(2):19. 10.3390/ijns9020019 10.3390/ijns9020019 PMC10123634 37092513

[23] Joseph R. Expanded newborn screening challenges to NICU nurses. Advances in Neonatal Care. 2017;17(3):151–161. | 10.1097/ANC.0000000000000381 10.1097/ANC.0000000000000381 28092316

[24] Khaton S. Effect of educational intervention on primary health care nurses’ knowledge regarding phenylketonuria disorder and PKU test. Nurs Health Sciences. 2016;5:45–58 10.9790/1959-0506014558

[25] Venegas C, Taljaard M, Reszel J, Harrison D. Barriers and facilitators to using pain treatment during newborn screening blood tests at a mother-baby unit. Nursing. 2019:139–44.

[26] Said H, Draz S. The effect of empowerment program for nurses regarding management of children with phenylketonuria. Evidence-Based Nurs Res. 2019;1(4). 10.47104/ebnrojs3.v1i4.107

[27] Khedr H, Al-Sharkawi S, Tantawi R. Assessment of nurses’ competency level regarding the care of infants with congenital anomalies of central nervous system. Egyptian Journal of Health Care. 2022;13(3).

[28] World Medical Association. World Medical Association Declaration of Helsinki. Ethical principles for medical research involving human subjects. Bull World Health Organ. 2001;79(4):373–4. 11357217 PMC2566407

