# Supplementary material for: Evaluation of primary health care nurses’ knowledge and neonatal screening performance for phenylketonuria in Alexandria
Source: BMC Nurs. 2025 Feb 7;24:145. doi: 10.1186/s12912-025-02719-4 (PMC11806625; doi:10.1186/s12912-025-02719-4)
Supplement: Supplementary file 1 — Supplementary Material 1 [file 12912_2025_2719_MOESM1_ESM.pdf]

Evaluation of Primary Health Care Nurses' Knowledge and Neonatal Screening Performance for Phenylketonuria in Alexandria

---

Tool

Tool one: Nurses' Knowledge Regarding Phenylketonuria Assessment Sheet:

Part I: Nurses' characteristics:

Nurse`s Age (years): …………

Educational level: Diplom ☐
Technical ☐
Bachelors ☐
Master ☐
PhD ☐

Years of experiences:………

Attendance of training courses about newborn Screening program for Phenylketonuria:
Yes ☐
No ☐

If yes ….
Number of training courses………………..
Duration of each course …………..

Part II: Nurses' knowledge Regarding Phenylketonuria Assessment Sheet: -

1. **What is the definition of phenylketonuria?**
   0- Don't know
   1- Correct and complete
   2- Correct and incomplete
   3- Wrong answer

2. What are the Causes of phenylketonuria?
   0- Don't know
   1- Lacks in the enzyme which is responsible for metabolizing the amino acid / change in the phenylalanine hydroxylase (PAH) gene.
   2- Other mention .

3. What are **the clinical manifestations for** phenylketonuria?
   0- Don't know
   1- musty odor from skin and urine
   2- Lighter skin, hair and eye color than family members

3- Seizures
4- Skin rashes /eczema
5- Tremors
6- hyperactivity.
7- Intellectual disability
8- Delayed development
9- Other mention

4. Which enzyme is missing in PKU?

0- Don’t know
1- Phenylalanine hydroxylase (PAH)
2- Other mention

5. What are the clinical manifestations that appear in neonates of PKU?

0- Don’t know
1- Low birth weight
2- Unusually small head
3- Problems with the heart
4- Other mention

6. What are the management of PKU?

0- Don’t Know
1- Dietary Therapy
2- Monitoring of Blood Phe &Tyr Levels
3- Taking vitamins, minerals and supplements.
4- Adding a supplemental medication called sapropterin dihydrochloride .
5- Other

9. What are the Potential Complications can be occurred?

0- Don’t know.
1- Delayed Developmental Milestones
2- Neurological problems
3- Behavioral, emotional, and social problems .
4- Irreversible brain damage and marked intellectual disability
5- Other mention

10. When the treatment of PKU disease should be started?

0- Don’t know
1- As soon after birth as possible
2- Other mention

11. When do Phenylketonuria symptoms start?

0- Don’t know.
1- In Infant stage from (1-23)months
2- Other mention

12. What is the concept of change in diets?

0- Don’t know.
1- Follow a low-phenylalanine (low protein diet)
2- Other mention

13. What are the severity of PKU?

0- Don’t know
1- Classic or severe PKU which means the enzyme needed to break down phenylalanine is missing or severely reduced.
2- Mild or moderate of PKU which means the enzyme still has some function
3- Other mention

14. What are Types of foods that should be restricted?

0- Don’t know.
1- Poultry
2- Meat
3- eggs
4- Cheese.
5- Fish.
6- Beef.
7- Beans.
8- Milk
9- Nuts
10- Chicken
11- According to doctor order

15. How long should the diet be used in PKU?

0- Don’t know
1- life-long dietary therapy.
2- Others, mentioned.

16 .What are Types of foods that should be allowed?

0- Don’t know.
1- The PKU diet includes foods that are low in protein, such as fruits and vegetables.
2- According to doctor order

17. What are Type of formula should be allowed?

- **0-** Don't know.
- **1-** Phenyl-Free 1 - iron-fortified infant formula.
- **2-** According to doctor order

18. How is PKU Diagnosed?

- **0-** Don't know.
- **1-** Routine Newborn screening via a blood test
- **2-** Other mention

19. Can breast milk be given to a PKU baby?

- **0-** Don't know
- **1-** Yes
- **2-** No
- **3-** Other mention

20. What is the normal level of phenylalanine in the blood?

- **0-** Don't know.
- **1-** Less than 2 mg/dL
- **2-** Other mention

21. What is the level of phenylalanine in the blood to be achieved during the treatment?

- **0-** Don't know.
- **1-** 2 and 6 mg/dL
- **2-** Other mention

22. When does the screening test should be done ?

- **0-** Don't know.
- **1-** In the first 48 to 72 hours of life.
- **2-** Other mention

23. What is the benefits of the screening test ?

- **0-** Don't know.
- **1-** Early detection and management to prevent irreversible brain damage.
- **2-** Other mention

24. What are the available community services to support children with phenylketonuria?

- **0-** Don't know.
- **1-** Family health center.
- **2-** Other mention

HEEL STICK (HEEL PUNCTURE)

STANDARD LIST OF EQUIPMENT & MEDICAL SUPPLIES

Steps	Done	Not done
1. Preform hand hygiene and clean blue tray.		
2. Collect equipment. Select the appropriate puncture device		
3. Administer 25% sucrose and provide comfort measures.		
4. Preform hand hygiene, apply gloves and prepare equipment.		
5. Nominate an area for puncture on the foot on the medial or lateral plantar surface (Picture 1). 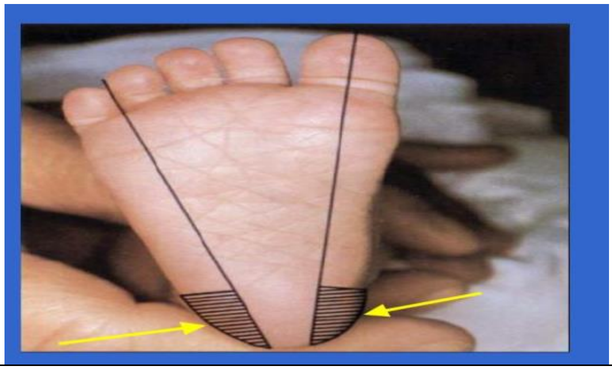		
6. Select the surface area to puncture. Continue in a “stepping” ladder pattern from the first puncture for subsequent blood sampling.		
7. Clean foot with alcohol and allow 30 seconds to dry completely. 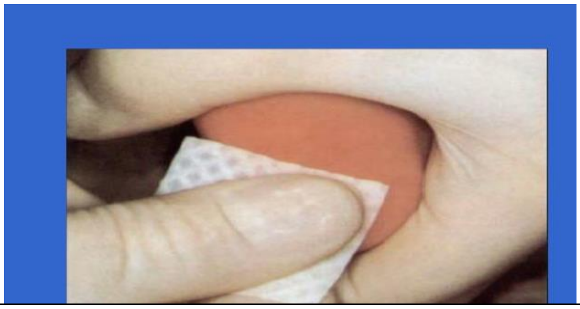		
8. Puncture heel holding the puncture device at a 90 degree angle (Picture 3) 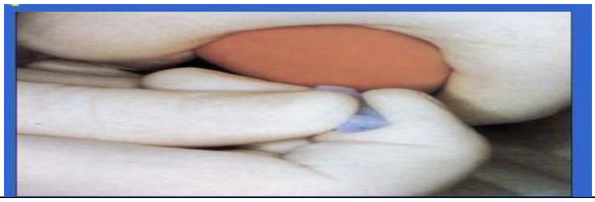		
9. Wipe away first drop of blood with gauze.		

<table>
<tr><td>10. Collect blood in correct Order of Draw (Picture 5), gently agitating tubes between each drop while avoiding scraping and scooping.
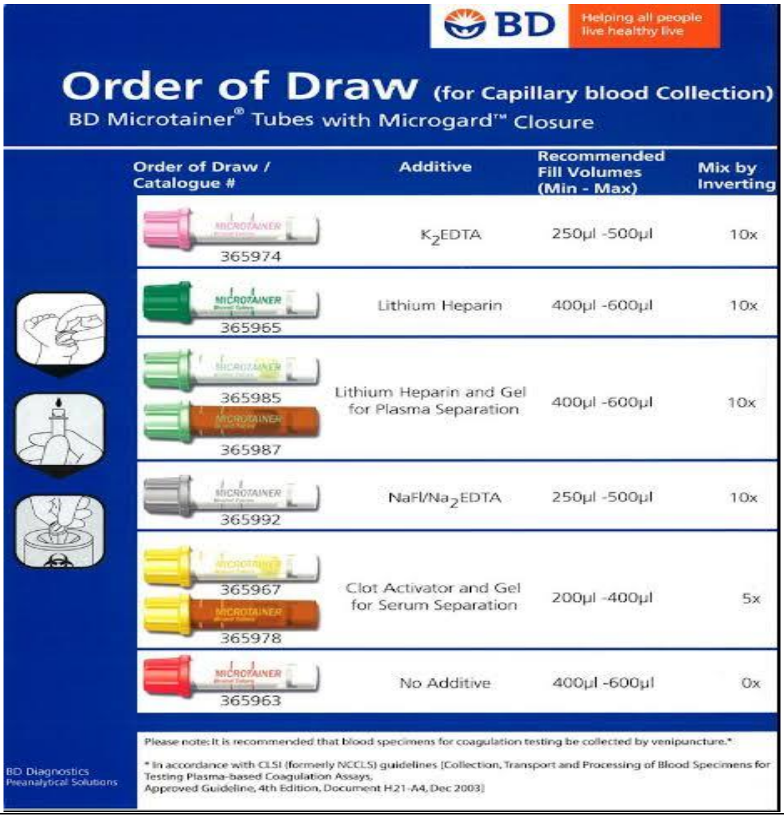
</td><td></td><td></td></tr>
<tr><td>11. Seal blood containers.</td><td></td><td></td></tr>
<tr><td>12. Apply direct pressure to puncture site.</td><td></td><td></td></tr>
<tr><td>13. Label collection tubes with correct the infant’s name label.</td><td></td><td></td></tr>
<tr><td>14. Dispose of puncture device in sharps container.</td><td></td><td></td></tr>
<tr><td>15. Collect and dispose of remaining equipment.</td><td></td><td></td></tr>
<tr><td>16. Clean blue tray and remove gloves.</td><td></td><td></td></tr>
<tr><td>17. Preform hand hygiene.</td><td></td><td></td></tr>
</table>
